# Postoperative demarcation lines in rhegmatogenous retinal detachment: what en-face imaging reveals about visual outcomes

**DOI:** 10.1007/s00417-026-07217-0

**Published:** 2026-03-27

**Authors:** Nicola Valsecchi, Lorenzo Padovani, Claudio Scardovelli Fava, Alessandro Finzi, Antonio Moramarco, Roberto dell’Omo, Luigi Fontana, Maurizio Mete

**Affiliations:** 1 Ophthalmology Unit, Dipartimento di Scienze Mediche e Chirurgiche, Alma Mater Studiorum University of Bologna, Bologna, Italy; 2 IRCCS Azienda Ospedaliero-Universitaria di Bologna, Bologna, Italy; 3 Department of Medicine and Health Sciences “Vincenzo Tiberio, ” University of Molise, Campobasso, Italy

**Keywords:** Retinal detachment, Demarcation lines, Perfluorocarbon liquid, Outer retinal alterations, Visual outcomes

## Abstract

**Purpose:**

To investigate postoperative demarcation lines using en-face optical coherence tomography (OCT) after pars plana vitrectomy (PPV) for primary rhegmatogenous retinal detachment (RRD) and assess association with visual outcomes.

**Methods:**

Consecutive patients undergoing PPV for primary RRD with gas tamponade were prospectively enrolled. Postoperative demarcation lines on en-face OCT were defined as hyper-reflective lines following contour of previous detachment edge. Postoperative best-corrected visual acuity (BCVA) and metamorphopsia were analyzed in relation to location of demarcation lines (within the central 3-mm ETDRS zone vs. between 3 and 6 mm). A control group of patients treated with silicone oil (SO) was included for comparison.

**Results:**

A total of 31 eyes met inclusion criteria. En-face OCT identified postoperative demarcation lines in 30 eyes (96.7%). None were seen in 25 eyes after SO removal (*p* < 0.001). Ellipsoid zone (EZ) disruption at demarcation line was found in 21 eyes, persistent subretinal fluid (SRF) in 6 eyes, and outer retinal folds (ORF) in 3 eyes. Eyes with persistent SRF had longer interval between symptom onset and surgery compared with those showing EZ disruption or ORF (*p* = 0.025). Demarcation lines within central 3-mm ETDRS zone were significantly associated with greater horizontal metamorphopsia (B = 0.232, *p* = 0.003), whereas no significant association was found with postoperative BCVA (B = − 0.094, *p* = 0.280).

**Conclusions:**

En face OCT accurately detects postoperative demarcation lines, which may represent a previously overlooked factor influencing visual dysfunction after PPV for RRD with gas tamponade.

## Key message

*****What is known?***:**

- Retinal demarcation lines have recently been reported following surgical repair of rhegmatogenous retinal detachment (RRD). These lines represent the boundary between regions of previously detached and reattached retina.
- However, the detailed characterization of the associated OCT features, the influence of various surgical approaches, and the clinical significance of these findings relative to their anatomical location remain incompletely understood.

*****What is new?***:**

- Demarcation lines following PPV with gas tamponade were almost ubiquitous on postoperative en face OCT imaging, whereas they were absent in eyes treated with silicone oil. Cross-sectional OCT analysis showed that ellipsoid zone disruption, subretinal fluid accumulation and outer retinal folds were observed along the course of demarcation lines.
- Notably, when these lines were within the central 3-mm ETDRS zone, they were associated with a higher incidence of horizontal metamorphopsia.
- These results suggest that demarcation lines might represent a previously overlooked factor influencing visual dysfunction following PPV with gas tamponade.

## Introduction

Rhegmatogenous retinal detachment (RRD) is the most common type of retinal detachment and occurs when a break in the neurosensory retina (NSR) allows vitreous fluid to enter the subretinal space, leading to separation of the NSR from the underlying retinal pigment epithelium (RPE) [1]. Despite high anatomical success rates following surgical repair, visual recovery—especially in fovea-involving cases—remains highly variable [2]. This discrepancy sparked growing interest in identifying factors that contribute to suboptimal visual outcomes after successful RRD reattachment.

The recovery of visual function post-RRD surgery is strongly influenced by the integrity of the photoreceptors. The degree of photoreceptor cell loss, retinal distortion, and subsequent remodeling of the outer retina significantly affects visual prognosis [3, 4]. Recent evidence underscores the importance of timely surgical intervention, as the duration of detachment is a critical determinant of functional outcomes [5, 6].

Cross-sectional optical coherence tomography (OCT) remains the standard imaging modality for evaluating outer retinal alterations. Specific post-operative OCT findings—such as ellipsoid zone (EZ) disruption, outer retinal folds, microcystoid macular edema, and persistent subretinal fluid—have been linked to poorer visual recovery [7–10]. However, some patients experience impaired visual outcomes despite unremarkable findings on conventional OCT, underscoring the limitations of current imaging approaches and the need for more advanced diagnostic tools. In this regard, en face OCT imaging offers a complementary perspective to conventional cross-sectional OCT by providing a top-down, laminar visualization of the retinal architecture. This technique may facilitate more precise localization of subtle outer retinal alterations following surgery.

Retinal demarcation lines have recently been described following surgical repair of RRD on en-face OCT images, with a reported prevalence of approximately 65% [11, 12]. These lines typically demarcate the border between previously detached and attached retina. Nonetheless, the detailed characterization of associated OCT findings, the effects of different surgical techniques employed, and the clinical implications of these features according to their anatomical localization remain poorly understood 13].

Therefore, the primary aim of this study was to evaluate the presence of postoperative demarcation lines using en-face OCT in a selected cohort of patients undergoing pars plana vitrectomy (PPV) with gas tamponade for primary RRD with partially detached macula (either fovea-off or fovea split). Furthermore, we compared the frequency of postoperative demarcation lines with that in a cohort of patients with primary RRD and partially detached macula who underwent PPV with silicone oil tamponade. A secondary objective was to investigate the relationship between the spatial localization of these demarcation lines and visual outcomes after surgery, as well as their morphological correlation with the duration of retinal detachment prior to intervention.

## Methods

### Study population

This prospective observational study included consecutive patients with primary RRD with partially detached macula (either fovea-off or fovea split) who underwent pars plana vitrectomy (PPV) with gas tamponade at the Ophthalmology Unit, University of Bologna, between June 2024 and February 2025.This study was approved by the Ethics Committee of Bologna, Italy (Cod CE: 53/2025/Oss/AOUBo). All procedures adhered to the principles of the Declaration of Helsinki, and all participants provided written informed consent.

Exclusion criteria were: tractional or exudative retinal detachment, PVR grade C or higher, epiretinal membrane or internal limiting membrane peeling during surgery, requirement for silicone oil tamponade, any primary treatment other than vitrectomy (e.g., pneumatic retinopexy or scleral buckling), absence or poor quality of pre- and postoperative macular OCT imaging, significant media opacities (excluding cataracts), presence of other macular diseases (such as intermediate or late-stage age-related macular degeneration), history of prior ocular surgery (except cataract surgery), axial length greater than 27 mm, and development of epiretinal membrane after surgery. All included patients underwent baseline evaluation and postoperative follow-up at 1, 3, and 6 months. A control cohort of patients with primary RRD and a partially detached macula who underwent PPV with silicone oil tamponade was also included for comparison.

### Ophthalmological assessment

At baseline and at each follow-up visit, patients underwent a comprehensive ophthalmological evaluation, including best corrected visual acuity (BCVA) measurement, slit lamp biomicroscopy, and intraocular pressure (IOP) assessment. A dilated fundus examination with a 90D indirect lens was performed to determine the location of the retinal break and the extent of the retinal detachment. SD-OCT imaging at baseline (Spectralis HRA-OCT; Heidelberg Engineering GmbH, Heidelberg, Germany) was carried out with two vertical and two horizontal single scans centered on the fovea, using automatic real-time (ART) values of 14 or higher, to confirm macular involvement and identify the border between the attached and detached retina. Baseline axial length was measured using the IOL Master 700 (Carl Zeiss Meditec, Jena, Germany). Postoperatively, metamorphopsia was objectively assessed using the M-CHARTS (Inami & Co., Ltd, Tokyo, Japan).

### Surgical technique

All patients underwent a standardized surgical procedure [14]. All surgeries were performed by two experienced surgeons (MM, AF). The choice between general and local anesthesia was determined based on patient preference and the anesthesiologist’s assessment, with local anesthesia administered via peribulbar nerve block. The procedures utilized 23-gauge PPV, and cataract extraction was performed concurrently when indicated.

During surgery, patients underwent a complete vitrectomy with peripheral vitreous shaving, and posterior hyaloid detachment from the retina was achieved if not present. At the surgeon’s discretion, triamcinolone acetonide (TA) was injected into the vitreous chamber to stain and identify any vitreous cortex remnants (VCR). If present, VCR were removed using a diamond-dusted membrane scraper, vitreous cutter, or microforceps and microscissors in a bimanual technique. Perfluorocarbon liquid (PFCL) was employed to reattach the retina in all cases, with subretinal fluid drained through peripheral retinal breaks during the fluid-air exchange. The procedure was finalized with endolaser photocoagulation around the retinal breaks and laser barricade on the peripheral retina. Intraocular air or gas was employed as a tamponade agent (SF6 or C3F8). In the control group, silicone oil 1000 cSt (Sil-1000, Dorc — Dutch Ophthalmic Research Center, Zuidland, The Netherlands), and heavy silicone oil (Densiron, Fluoron GmbH, Ulm, Germany) were used according to the surgeon’s discretion. Postoperative positioning was determined based on the location of the retinal breaks. In the case of superior breaks, the patient was advised to maintain a semi-sitting position for the first postoperative day. In the case of inferior breaks, however, a supine position was recommended. Following surgery, patients were prescribed a tapering regimen of topical antibiotics and steroids for 4 weeks. In the control group, silicone oil was removed after 3 to 6 months from the first surgery.

### Post-operative OCT images acquisition

All subjects underwent a standardized SD-OCT imaging protocol, which included infrared (IR) imaging, fundus autofluorescence (FAF), and a dense 30° × 25° raster scan composed of 241 B-scans spaced 30 μm apart. En-face images were generated through three-dimensional reconstruction using the device’s proprietary software, and the reference slabs were aligned to the level of photoreceptors inner segment/outer segment.

A retinal demarcation line was defined as a postoperative hyper-reflective line visible on enface. It was situated between an area of alterations of the outer retinal layers - consistent with previously detached retina—and adjacent normal outer retina. These lines had to correspond to the border of the attached and detached retina on pre-operative OCT. Two independent masked graders (NV, LP) evaluated the en-face OCT images at 1 month, 3 and 6 months, and any segmentation errors were manually corrected using the device’s built-in correction tools.

Presence of demarcation lines was determined based on consensus between the two graders; in cases of disagreement, a third grader (MM) made the final decision. Similarly, fading of demarcation lines from 1 to 3 months to 6 months was independently evaluated by the same two graders, with any discrepancies resolved by the third grader.

Furthermore, the two graders (LP, NV) evaluated IR images for the presence of demarcation lines, blinded to each other’s assessments and clinical data. Evaluation from the first grader (LP) was used for the analysis, whereas the assessment from the second grader (NV) was used for inter-rater reliability analysis.

Corresponding OCT scans at the sites of demarcation lines were further analyzed to characterize associated retinal alterations. Disruption of the inner segment ellipsoid zone (EZ) was defined as an interruption or discontinuity of the ellipsoid band. Persistence of subretinal fluid (SRF) was identified by the presence of SRF in the absence of outer retinal folds (ORF). Additionally, the presence of microcystoid macular edema (MME) was assessed according to previously established criteria [15]. To evaluate the association between demarcation line localization and visual outcomes, an ETDRS grid was overlaid on the en face OCT images, using the device’s proprietary software. The localization of each demarcation line was classified according to whether it intersected the central 3 mm from the fovea or was located between 3 and 6 mm. Also, the presence of retinal vessel printings (RVP) was assessed by two independent graders (LP, NV), as previously described [16, 17]. The presence of RVPs was taken as evidence of retinal displacement. When there was disagreement between the two graders, a third grader (MM) made the final decision.

### Statistical analysis

Demographic and clinical data were reported using descriptive statistics, with appropriate measures of central tendency and variability depending on the nature of the variable (categorical or continuous). The Shapiro–Wilk test was employed to assess the normality of distribution for continuous variables. As variables were not normally distributed, they were reported as median and interquartile ranges (IQR), and non-parametric tests were used in the statistical analysis. Associations between categorical variables were evaluated using Fisher’s exact test. McNemar’s test was utilized to compare detection rates of demarcation lines between en face OCT and IR imaging. The inter-rater reliability was measured by the absolute agreement model of the intra-class correlation coefficient (ICC). The Mann-Whitney test was used to compare visual parameters in relation to the localization of demarcation lines. Multivariate generalized linear models were conducted to evaluate the independent associations between clinical variables and M-CHARTS horizontal and vertical, as well as BCVA. A p-value of < 0.05 was considered indicative of statistical significance. All analyses were performed using IBM SPSS Statistics, version 26.

## Results

### Demographic data

**Fig. 1:**
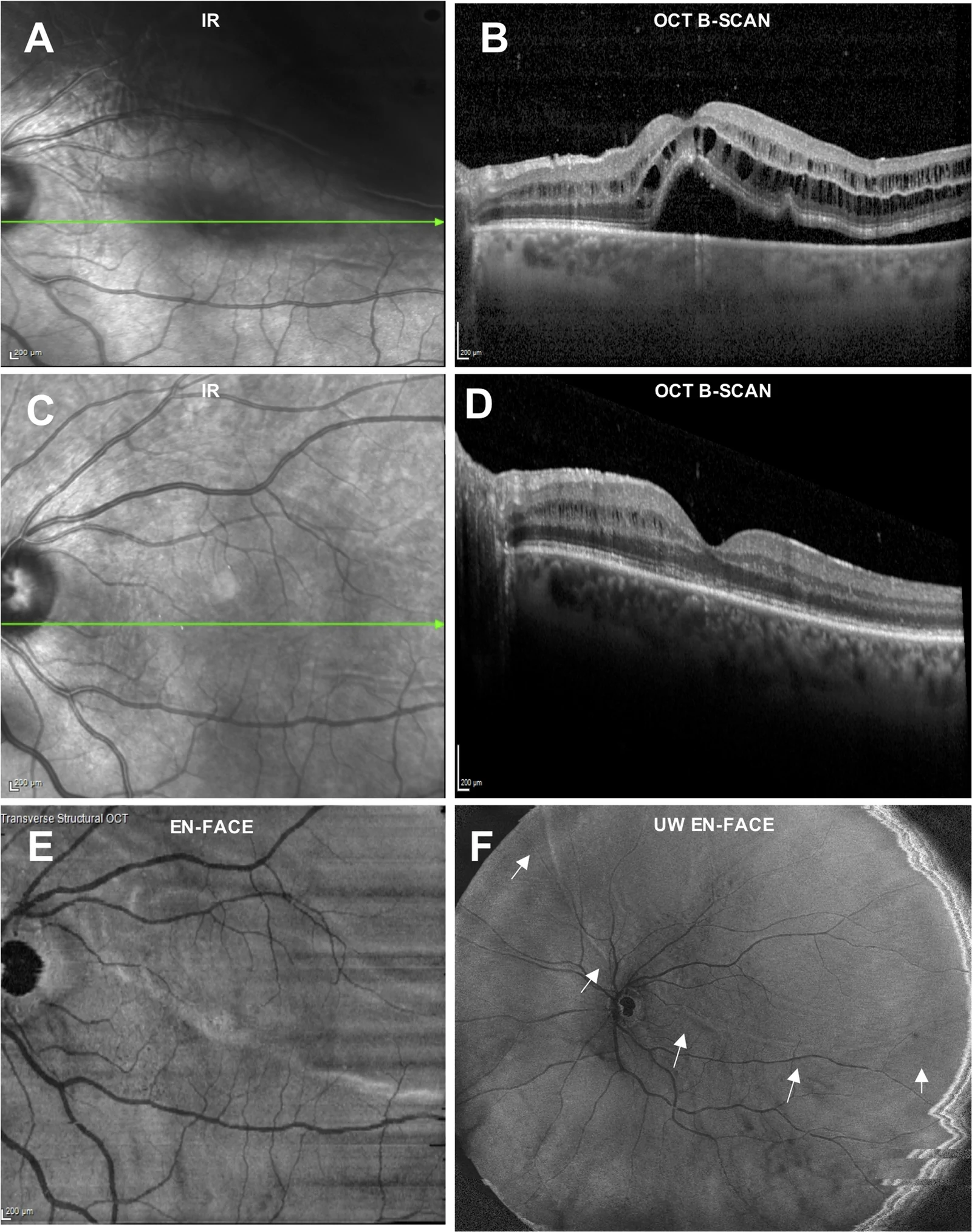
Representative example of postoperative demarcation lines. **A–B)** Baseline infra-red (IR) and optical coherence tomography (OCT B-scan) of a 65-year-old female presenting with primary superior rhegmatogenous retinal detachment involving the fovea and two superotemporal retinal breaks in the left eye. **C–D)** IR and cross-sectional OCT images acquired 4 months after pars plana vitrectomy with sulfur hexafluoride (SF₆) gas tamponade. IR imaging shows no evident alterations. However, cross-sectional OCT reveals disruption of the ellipsoid zone at the fovea and in the temporal macula, along with microcystoid macular edema in the perifoveal nasal region. **E)** En-face spectral-domain OCT of the same eye demonstrates a hyperreflective demarcation line within the central 3-mm ETDRS zone, which is not visible on the corresponding IR image. **F)** Ultra-widefield en-face OCT (UW-en face) highlights the demarcation line delineating the extent of the previously detached retina (white arrows)

**Fig. 2:**
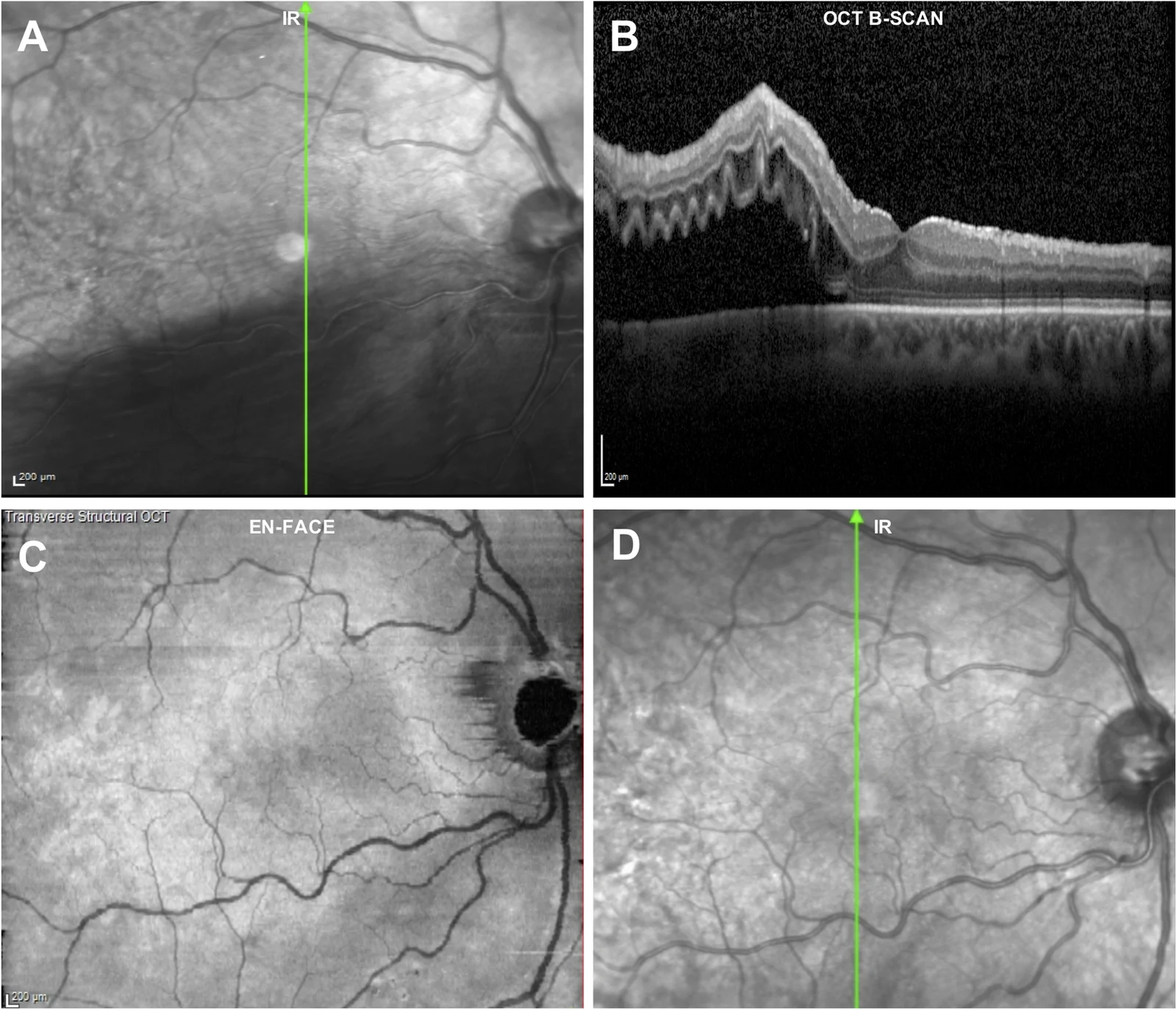
Representative example of post-operative en-face optical coherence tomography (OCT) after silicone oil removal. **A–B)** Baseline optical coherence tomography (OCT) of a 75-year-old female presenting with primary inferior rhegmatogenous retinal detachment foveal split, and three inferior retinal breaks in the right eye. Patient underwent pars plana vitrectomy with silicone oil tamponade (silicone oil 1000 cSt).**C) and D)** After 4 months from surgery and 13 days from silicone oil removal, en-face spectral-domain OCT and infrared images did not show any demarcation lines

During the study period, a total of 145 surgeries for RRD were performed, including 69 eyes treated with PPV and gas tamponade for primary RRD. Among them, 38 eyes were excluded. Reasons for exclusion were axial length greater than 27 mm (*n* = 13), fovea-on RRD (*n* = 12), poor quality of pre-operative OCT (*n* = 6), epiretinal membrane and/or internal limiting membrane peeling during surgery (*n* = 4), concomitant age-related macular degeneration (*n* = 2), and previous corneal transplant (*n* = 1). As a result, 31 eyes met the inclusion criteria. In this subgroup of 31 eyes, postoperative demarcation lines were observed in 30 eyes (96.7%) using en-face OCT imaging (Fig. 1). Conversely, IR imaging detected these lines in only 12 of the 30 eyes (40.0%) (*p* < 0.001). The measurements by both readers showed a good agreement for IR images (ICC = 0.889, CI 0.719–0.956), and an excellent agreement for en-face OCT images (ICC = 0.975, CI 0.940–0.998). As a control group, 25 eyes from 25 patients who underwent PPV with silicone oil tamponade for primary RRD were included. None of these eyes exhibited visible demarcation lines on either en-face OCT or IR imaging (*p* < 0.001) after silicone oil removal. See Fig. 2. A post-hoc power assessment using a two-proportion z-test approximation, based on the observed demarcation-line rates (96.7% vs. 0%), indicated that even a sample of 10 eyes per group would provide > 80% power at α = 0.05, confirming that the current cohort size is adequate to support the primary study outcome. Baseline demographic and clinical data of patients included are presented in Table 1. In the gas tamponade group, SF6 was used in 90% of the cases, air in 6.6%, and C3F8 in 3.3%. In the silicone oil group, silicone oil 1000 cSt was used in 80% of the cases, and Densiron in 20%, and silicone oil removal was performed after a median of 4.1 months (3.1–5.3) from the first surgery.

**Table 1:** Demographic data are reported

Demographic data	Gas tamponade group (*n* = 31)	Silicone oil group (*n* = 25)	*P* value
Age, median (IQR)	60.5 (55–68.2.2)	61.2 (56.1–70.4)	0.856
Sex, female, n (%)	10 (32.3%)	12 (48%)	0.675
Pseudophakia, n (%)	10 (32.2%)	10 (40%)	0.583
Fovea-off, n (%)	25 (80.6%)	20 (80%)	0.901
Fovea-split, n (%)	6 (19.3%)	5 (20%)	0.922
Pre-op BCVA, median (IQR)	0.5 (0.4–1)	0.6 (0.5–1)	0.756
Symptoms to treatment, hours, median (IQR)	96 (72–168)	102 (70–210)	0.564
Diagnosis to treatment, hours, median (IQR)	24 (14–60)	26 (15–72)	0.465
Retinal breaks, number, median (IQR)	2.4 (1.5–6.5)	3.2 (1.7 −8)	0.256
PVR grade, n (%) - Grade A - Grade B	15 (48.3%) 3 (9.7%)	15 (60%) 6 (24%)	0.567 0.098
Location of retinal detachment, n (%)			
- Superior	22 (70.9%)	13 (52%)	0.153
- Inferior	9 (29%)	12 (48%)	0.263
- Temporal	3 (9.7%)	0	0.057
Post-op BCVA, median (IQR)	0.1 (0.03–0.2)	0.2 (0.1–0.3)	0.125
Demarcation lines, n (%)	30 (96.7%)	0	**< 0.001**
Retinal displacement, n (%)	14 (45.1%)	8 (32%)	0.354
Microcystoid macular edema, n (%)	6 (19.3%)	5 (20%)	0.876

IQR=interquartile range. BCVA=best corrected visual acuity. PVR=proliferative vitreoretinopathy.

### Post-operative demarcation lines and outer retinal alterations

**Fig. 3:**
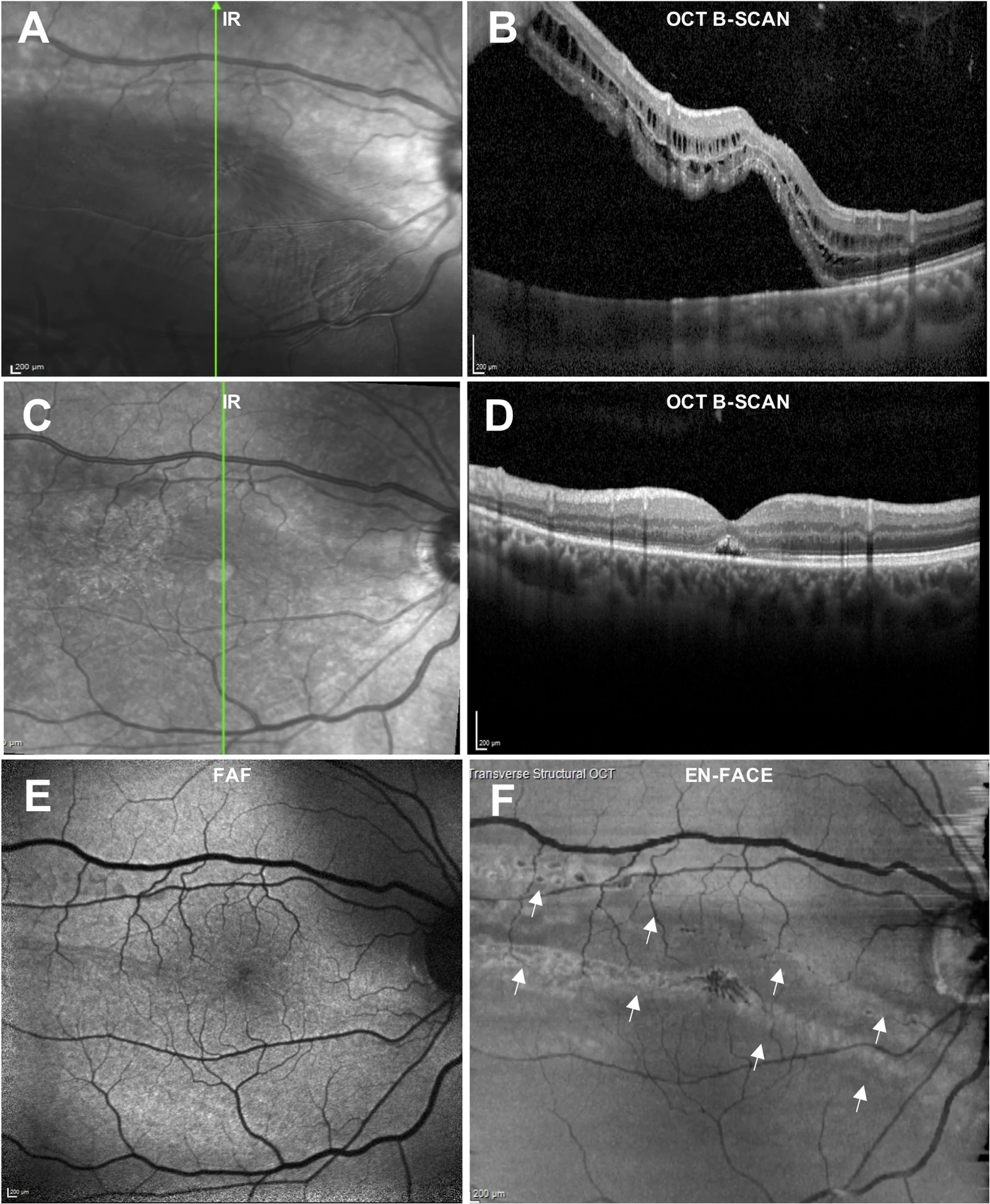
Two demarcation lines in the same eye with inferior retinal detachment. **A–B)** Baseline optical coherence tomography (OCT) of a 58-year-old male presenting with primary inferior rhegmatogenous retinal detachment (RRD) involving the fovea and one inferior retinal break in the right. The patient had been experiencing symptoms for 14 days but had not presented to the Ophthalmology Department. **C)** Infra-red (IR) imaging acquired 5 months after pars plana vitrectomy (PPV) with sulfur hexafluoride (SF₆) gas tamponade shows no evident alterations. **D)** Cross-sectional OCT demonstrates the presence of ellipsoid zone disruption and persistence of subretinal fluid (SRF) under the fovea. **E)** Fundus autofluorescence (FAF) reveals areas of hypo-autofluorescence in the macula, although no clear demarcation lines are visible. **F)** En-face spectral-domain OCT reveals two hyperreflective demarcation lines: one above the fovea and another within the central 3-mm ETDRS zone (white arrows). Note the areas of hyporeflectivity corresponding to the accumulation of SRF

**Fig. 4:**
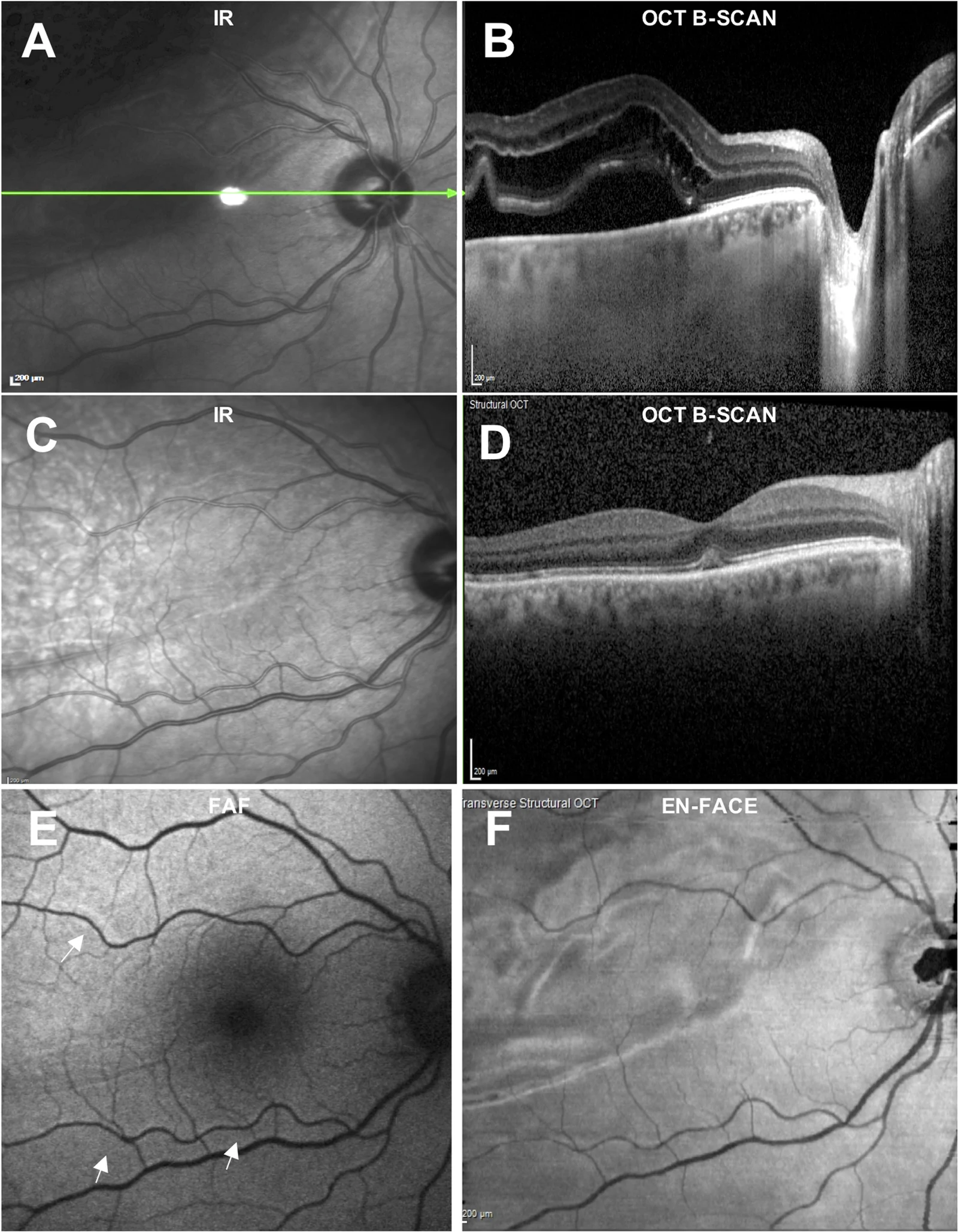
Post-operative demarcation line in a patient with superior retinal detachment. **A–B)** Baseline optical coherence tomography (OCT) of a 58-year-old male presenting with primary superior rhegmatogenous retinal detachment foveal split and three superotemporal retinal breaks in the right eye. **C)** Infra-red (IR) imaging acquired 6 months after pars plana vitrectomy (PPV) with sulfur hexafluoride (SF₆) gas tamponade shows a demarcation line within the central 3-mm ETDRS zone. **D)** Cross-sectional OCT demonstrates the presence of an outer retinal fold under the fovea. **E)** Fundus autofluorescence (FAF) reveals a hypo-autofluorescent line corresponding to the demarcation line within the central 3-mm ETDRS zone. Also, retinal displacement can be observed (white arrows). **F)** En-face spectral-domain OCT clearly demonstrate the demarcation line defining the boundaries between previously attached and detached retina

One demarcation line was observed in 29/30 (96.7%), whereas two demarcation lines were observed in 1/30 (3.3%) eye. See Fig. 3. In 90% of cases, the demarcation lines exhibited a horizontal orientation within the central 3-mm ETDRS zone, whereas only 10% showed a vertical orientation. In 70% of eyes, demarcation lines exhibited partial fading between the 1- and 3-month visits and the 6-month follow-up, although they remained clearly visible in all cases at 6 months. Overall, 21/30 (70%) eyes demonstrated EZ disruption at the site of the demarcation lines, 6/30 (20%) eyes demonstrated EZ disruption with persistent SRF adjacent to the course of the demarcation lines, and 3/30 (10%) eye showed the presence of an ORF at the site of the demarcation line. See Fig. 4. Fundus autofluorescence imaging did not reveal a demarcation line in 23 eyes; however, in 4 eyes and in 3 eyes, the demarcation line appeared hyperautofluorescent and hypoautofluorescent, respectively. When hypoautofluorescent, persistent SRF was present in 2 out of 3 eyes, likely indicating masking by persistent SRF or early RPE atrophy. On the other hand, when hyperautofluorescent, EZ disruption alone was observed at the cross-sectional OCT, likely indicating concomitant RPE stress. Retinal displacement, as evidenced by the presence of RVPs, was observed in 14/30 (46.7%) eyes. MME was identified in 6 eyes and was not located along the course of the demarcation lines. Eyes with EZ disruption and persistent SRF adjacent to the location of the demarcation lines had a longer interval between symptom onset and surgical intervention (median = 12.5, IQR = 4–22.5.5 days) compared to those with EZ disruption alone or ORF (median = 4, IQR = 3–6.5 days) (*p*=0.0.025).

### Demarcation lines and visual outcomes

Overall, 18 eyes exhibited demarcation lines within the central 3-mm ETDRS zone, while in 12 eyes, the demarcation lines were located between 3 and 6 mm. Horizontal metamorphopsia was significantly greater in eyes with demarcation lines within the central 3-mm ETDRS zone compared to those with demarcation lines between 3 and 6 mm. In contrast, there were no significant differences between the two groups in BCVA or vertical metamorphopsia (Table 2**).** In the multivariate analysis, longer time from symptom onset to treatment was significantly associated with post-operative worse BCVA. (Table 3**).** Furthermore, longer time from symptom onset and diagnosis to treatment were both independently associated with greater horizontal metamorphopsia (B = 0.001, *p* < 0.001, B = 0.003, *p* = 0.002 respectively**)**. Also, foveal EZ disruption without SRF and foveal demarcation lines within the central 3-mm ETDRS zone were associated to higher horizontal metamorphopsia (B = 0.246, *p* = 0.004 B = 0.232, *p* = 0,003, respectively) compared to eyes with EZ disruption and SRF or ORF and eyes with demarcation lines between 3 and 6 mm, respectively. See Table 4. On the other hand, no clinical and OCT variables were significantly associated with vertical metamorphopsia (*p* > 0.05). See Table 5.

**Table 2:** Post-operative visual outcomes based on the location of demarcation lines

	Demarcation lines ≤ 3 mm from foveal center (*n* = 18)	Demarcation lines > 3 mm from foveal center (*n* = 12)	*P* value
BCVA, LogMAR	0.1 (0–0.2.2)	0.2 (0–0.2.2)	0.532
Vertical metamorphopsia	0.3 (0.2–0.4)	0 (0–0.5.5)	0.195
Horizontal metamorphopsia	0.3 (0–0.5.5)	0 (0–0.1.1)	**0.044**

BCVA=best corrected visual acuity. LogMAR=logarithm of the Minimum Angle of Resolution. The Mann-Whitney test was used for statistical analysis

**Table 3:** Multivariate analysis of clinical and optical coherence tomography (OCT) features associated with post-operative best-corrected visual acuity (BCVA)

	B	95% Confidence interval		*p*-value
Age (years)	0.002	−0.003	0.007	0.483
Symptoms to Treatment (hours)	0.002	0.001	0.003	**0.016**
Diagnosis to Treatment (hours)	0.000	−0.001	0.001	0.315
EZ disruption alone (yes/no)	−0.101	−0.219	0.018	0.296
Retinal Displacement (yes/no)	0.131	0.028	0.234	0.313
Demarcation lines within 3 mm (yes/no)	−0.094	−0.200	0.011	0.280

The table presents the B coefficients, 95% confidence intervals. A p-value of less than 0.05 was considered statistically significant, with significant p-values highlighted in bold. B = regression coefficient, EZ=ellipsoid zone. Generalized linear models were used for multivariate analysis

**Table 4:** Multivariate analysis of clinical and optical coherence tomography (OCT) features associated with post-operative horizontal metamorphopsia

	B	95% Confidence interval		*p*-value
Age (years)	0.006	−0.002	0.013	0.134
Symptoms to Treatment (hours)	0.001	0.001	0.002	**< 0.001**
Diagnosis to Treatment (hours)	0.003	0.001	0.004	**0.002**
EZ disruption alone (yes/no)	0.246	0.077	0.415	**0.004**
Retinal Displacement (yes/no)	0.120	−0.036	0.277	0.132
Demarcation lines within 3 mm (yes/no)	0.232	0.077	0.388	**0.003**

The table presents the B coefficients, 95% confidence intervals. A p-value of less than 0.05 was considered statistically significant, with significant p-values highlighted in bold. B = regression coefficient, EZ=ellipsoid zone. Generalized linear models were used for multivariate analysis

**Table 5:** Multivariate analysis of clinical and optical coherence tomography (OCT) features associated with post-operative vertical metamorphopsia

	B	95% Confidence interval		*p*-value
Age (years)	0.001	−0.016	0.018	0.926
Symptoms to Treatment (hours)	0.000	−0.001	0.001	0.598
Diagnosis to Treatment (hours)	0.003	−0.001	0.007	0.145
EZ disruption alone (yes/no)	0.142	−0.257	0.541	0.485
Retinal Displacement (yes/no)	−0.035	−0.404	0.335	0.853
Demarcation lines within 3 mm (yes/no)	0.050	−0.317	0.416	0.791

The table presents the **B** coefficients, 95% confidence intervals. A p-value of less than 0.05 was considered statistically significant, with significant p-values highlighted in bold. B = regression coefficient, EZ=ellipsoid zone. Generalized linear models were used for multivariate analysis

## Discussion

The primary finding of the present study was that en-face OCT imaging detected postoperative demarcation lines in approximately 95% of eyes treated with PPV and gas tamponade for primary RRD and partially detached macula, whereas no demarcation lines were observed in eyes treated with silicone oil tamponade. Also, multivariate analysis showed that eyes in which demarcation lines were within the central 3-mm ETDRS zone exhibited more pronounced horizontal metamorphopsia compared to those with demarcation lines located between 3 and 6 mm.

Demarcation lines were first described by Thomseth et al. using en face swept-source OCT in approximately 63% of eyes following RRD repair [11]. The study included eyes that had undergone both scleral buckle and vitrectomy; however, it lacked detailed information regarding the surgical techniques employed. A subsequent study by the same group reported macular demarcation lines in 91% of eyes with OCT-confirmed partial macular detachment following various surgical techniques and tamponade agents. Although no association was found with postoperative BCVA, metamorphopsia, or aniseikonia, the study did not assess the anatomical localization of the demarcation lines in relation to visual outcomes [13]. Recently, Brosh et al. described the characteristics of demarcation lines on IR imaging in eyes treated with PPV and pneumatic retinopexy (PnR). Twenty-four eyes were treated with gas, and only one eye was treated with silicone oil tamponade. In their study, all demarcation lines gradually faded over time, becoming undetectable on IR-OCT after a mean duration of approximately three months [12]. In the present study, en face OCT identified postoperative demarcation lines as an almost ubiquitous finding still visible at 6 months postoperatively in eyes treated with PPV and gas tamponade, and it proved to be more sensitive than IR imaging in detecting these alterations. On the other hand, we did not observe any demarcation lines in eyes treated with silicone oil. These findings underscore the importance of postoperative en face OCT imaging for the assessment of demarcation lines and suggest that these alterations are mainly present in eyes treated with PPV with gas tamponade. Furthermore, we observed a partial fading of demarcation lines between the 1- and 3-month visits and the 6-month follow-up in 70% of the cases, suggesting that structural recovery may occur over time.

Another finding of the present study was that greater horizontal metamorphopsia was independently associated with the presence of demarcation lines within the central 3-mm ETDRS zone, a longer interval between symptom onset and treatment, and foveal EZ disruption. Additionally, delayed treatment following symptom onset was significantly correlated with poorer post-operative BCVA. Previous studies reported that a prolonged interval between symptom onset and surgical intervention negatively affects visual outcomes [9]. In a recent meta-analysis, Sothivannan et al. showed that early repair of macula-off RRD may lead to better final BCVA compared to delayed repair [18]. Regarding post-operative metamorphopsia, previous studies reported a higher degree of metamorphopsia in eyes with post-operative EZ disruption [10, 19]. On the other hand, the role of retinal displacement on post-operative visual outcome remains controversial [20]. Brosh et al. observed a higher prevalence of vertical metamorphopsia in eyes with displacement [21]. In contrast, Kumar et al. found no significant relationship between retinal displacement and the severity of metamorphopsia [22]. In the present study, we did not find any association between retinal displacement and postoperative metamorphopsia or visual acuity. On the other hand, a higher degree of metamorphopsia observed in eyes with demarcation lines located within the central 3-mm ETDRS zone suggests that localization of demarcation lines might contribute to persistent visual disturbances, even after anatomically successful retinal reattachment. Therefore, demarcation lines might represent a previously overlooked factor influencing visual dysfunction following PPV for RRD. Interestingly, this effect was directionally dependent, as only horizontal metamorphopsia showed a significant association with centrally located demarcation lines. This may reflect the predominance of horizontally oriented alterations along the former detachment edge, implying that the spatial orientation of demarcation lines might influence the specific axis of postoperative visual disturbance. Therefore, future research comparing eyes with horizontally versus vertically oriented demarcation lines is needed to determine whether orientation-specific structural changes consistently correspond to the direction of metamorphopsia.

Our cross-sectional OCT analysis showed that EZ disruption was consistently present at the site of demarcation lines. Furthermore, in 20% of cases it was accompanied by SRF accumulation and in 10% of the cases by ORF. Photoreceptors have a high metabolic demand, and their separation from the RPE during detachment results in hypoxia and cellular swelling, which disrupt photoreceptor integrity. To regain optimal function after retinal detachment repair, they must recover not only from the hypoxic and metabolic insult, but also restore their close alignment and spacing with RPE [23]. While disruption of the EZ primarily reflects damage to the mitochondria-rich region within the outer portion of the photoreceptor inner segments, accumulation of SRF is mainly caused by impaired fluid reabsorption due to RPE dysfunction [24]. On the other hand, ORFs are persistent folds that form when outer retinal corrugations (ORCs) fail to resolve before the retina reattaches. Formation of ORCs occurs when liquified vitreous overwhelms RPE regulation during RRD, leading to hydration-induced lateral expansion of the outer retina [25]. Interestingly, eyes with EZ disruption and persistent SRF had a longer interval between symptom onset and surgical intervention compared to those with EZ disruption alone or ORF, suggesting that a prolonged interval between symptom onset and surgical intervention may exacerbate RPE pump failure, leading to impaired fluid reabsorption and persistent SRF accumulation. Overall, our findings indicate that the demarcation border between previously attached and detached retina constitutes a particularly vulnerable anatomical region. Additional studies are required to determine whether the different cross-sectional alterations observed arise from distinct pathogenic mechanisms or represent variable stages of a single pathophysiological process driven by photoreceptor injury and RPE pump dysfunction at this fragile site.

The preferential localization of retinal alterations at the junction between previously detached and attached retina may result from a combination of factors related both to the detachment itself and the surgical technique employed. In the detached retina, SRF accumulation may increase shear and tractional forces at the demarcation line. Also, SRF acts as an unstirred layer, reducing oxygen absorption by photoreceptors in the detached area and limiting oxygen flux from the choroid to the inner segments, leading to local oxygen accumulation [23]. The resulting increase in oxygen availability at this border may trigger oxidative stress, contributing to photoreceptor and RPE damage. Additionally, this transitional zone may serve as a focus of localized inflammation and glial activation, further promoting tissue remodeling and photoreceptor degeneration. Overall, these mechanisms likely render the junction between previously detached and attached retina a highly vulnerable anatomical site, increasing its susceptibility to postoperative alterations.

**Fig. 5:**
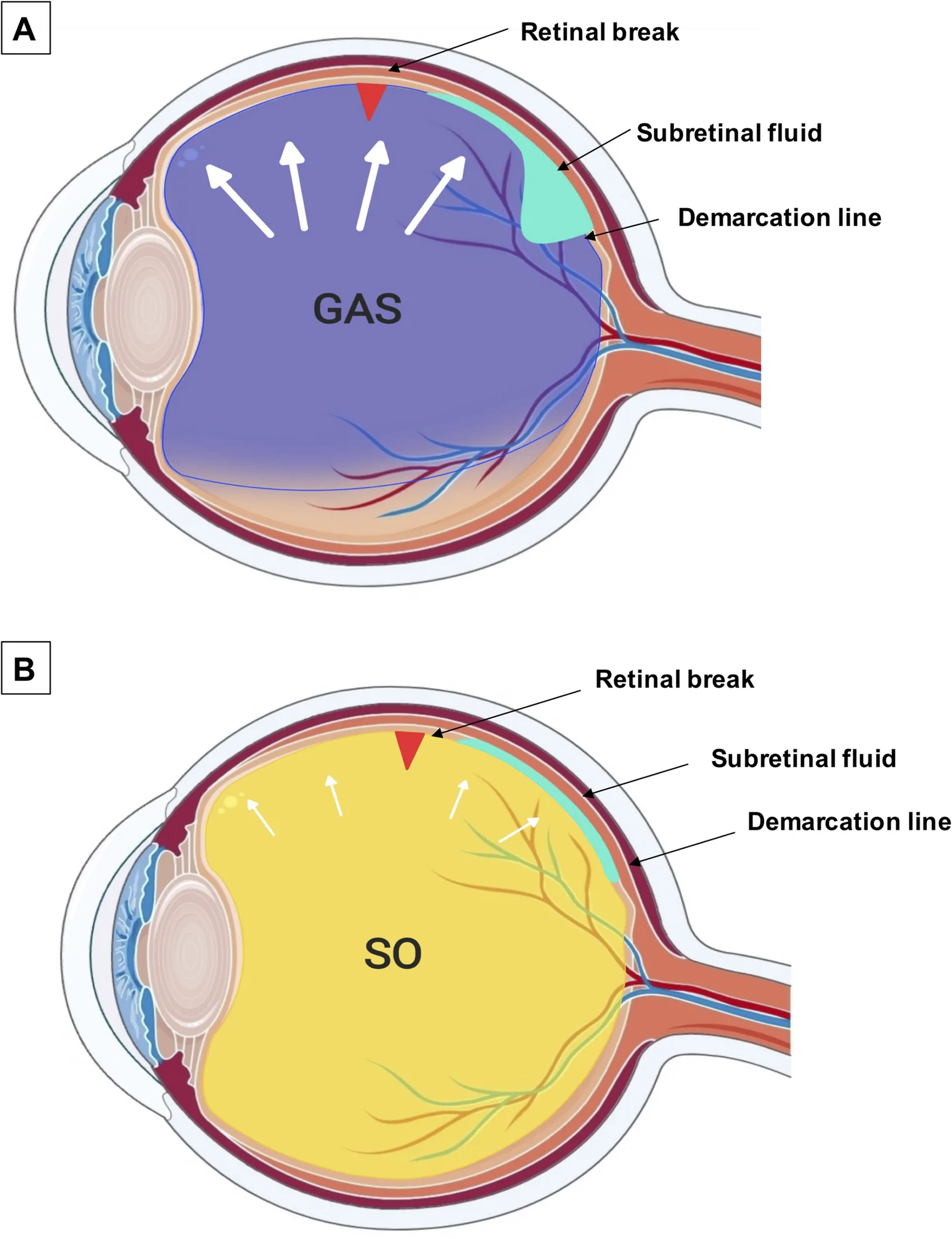
Effects of gas tamponade and silicone oil on the formation of demarcation lines after pars plana vitrectomy for rhegmatogenous retinal detachment. **(A)** The high buoyancy (white arrows) and low viscosity of intraocular gas, combined with postoperative positioning, promote the displacement of residual subretinal fluid (SRF) toward the macular margin of the previous detachment. In this region, the retinal pigment epithelium (RPE) is subjected to increased SRF volume, while the retina experiences elevated shear stress and disrupted retinal–RPE interactions. These mechanical and metabolic stresses may underlie the higher incidence of ellipsoid zone (EZ) alterations along the demarcation line, the persistence of SRF in chronic detachments due to RPE pump dysfunction, and the formation of outer retinal folds (ORF). **(B)** In contrast, silicone oil, with lower buoyancy (white arrows) and a more uniform tamponade effect, does not induce directional fluid shifts. This reduces localized mechanical stress at the detachment margin, which may explain the lower prevalence of demarcation lines following silicone oil removal

Furthermore, the use of gas tamponade may influence the formation of demarcation lines after surgery. The high buoyancy and low viscosity of intraocular gas, combined with postoperative positioning, actively displace residual SRF toward the macular edge of the previous detachment. In this region, the RPE must handle an increased volume of SRF, while the retina is subjected to elevated shear stress and altered retinal–RPE interactions. These mechanical and metabolic stresses may contribute to the higher frequency of EZ alterations observed at the demarcation line, as well as to a greater risk of persistent SRF in longer-standing detachments due to RPE pump failure and the development of ORF. In contrast, silicone oil, with its lower buoyancy and more uniform tamponade effect, does not generate the same directional fluid shifts and therefore attenuates these localized stresses and retinal remodeling at the detachment edge. See Fig. 5.

Also, PFCL was used intraoperatively in all cases to achieve retinal reattachment. Owing to its high specific gravity and surface tension, PFCL can exert localized mechanical forces at the edge of the detached retina, potentially increasing stress at the reattachment interface and promoting misalignment between photoreceptors and the RPE [26]. Additionally, fluid–air exchange may impose further mechanical stress on the photoreceptor–RPE interface at the demarcation line. The drainage technique employed during PPV had been shown to influence long-term anatomical and functional outcomes. Specifically, PFCL-assisted drainage was associated with poorer visual acuity and a higher risk of outer retinal band disruption compared to drainage via a peripheral retinal break or posterior retinotomy [27]. Thus, by promoting rapid retinal flattening, PFCL may increase the incidence of demarcation lines and be associated with more pronounced structural changes and greater horizontal metamorphopsia [27]. Overall, our findings underscore the need for further research to elucidate the mechanisms underlying demarcation line formation and to assess their impact on visual outcomes across various surgical approaches. As gas tamponade represents the preferred approach in uncomplicated primary RRD, understanding mechanisms behind demarcation lines formation could guide vitreo-retinal surgeons in minimizing demarcation line formation.

The main limitation of this study is the limited sample size, which may affect the generalizability of the findings. Nonetheless, strict inclusion and exclusion criteria were applied to minimize confounding variables and to ensure a well-defined and homogeneous cohort. Additionally, the relatively short duration of follow-up restricts the ability to fully characterize the temporal dynamics and potential progression of demarcation lines. Consequently, larger prospective studies with extended longitudinal follow-up are warranted to comprehensively assess the natural history of these structural changes and their sustained impact on visual outcomes.

## Conclusions

In the present study, demarcation lines following PPV with gas tamponade for macula-off primary RRD were almost ubiquitous on postoperative en face OCT imaging, whereas they were absent in eyes treated with silicone oil. Notably, when these lines were within the central 3-mm ETDRS zone, they were associated with a higher incidence of horizontal metamorphopsia. These results suggest that demarcation lines might represent a previously overlooked factor influencing visual dysfunction following PPV with gas tamponade. Accordingly, en-face OCT should be incorporated as a standard imaging modality in the postoperative follow-up of RRD patients. Furthermore, further research is warranted to elucidate the underlying mechanisms of demarcation line formation and to evaluate their long-term impact on visual function across different surgical techniques.

## Data Availability

Data cannot be shared openly but are available on request from authors.
